# From skepticism to trust: cultivating confidence in pandemics

**DOI:** 10.1097/MS9.0000000000003827

**Published:** 2025-09-05

**Authors:** Ifeanyichukwu Akuma, Vina Vaswani

**Affiliations:** a Centre for Ethics, Yenepoya Medical College, Yenepoya (deemed to be) University, Mangalore, Karnataka, India; b Centre for Ethics, Yenepoya Medical College, Yenepoya (deemed to be) University, Mangalore, Karnataka, India

**Keywords:** community engagement, public health strategies, vaccine communication, vaccine education, vaccine policy

## Abstract

Vaccine hesitancy presents a significant challenge to public health efforts, particularly during pandemics. This article complies with the TITAN 2025 guideline^[1]^ and explores a structured approach to transforming skepticism into trust by integrating a framework such as the “Trust-Engagement-Policy” (TEP) model alongside region-specific scenarios. Transparent communication, culturally sensitive community engagement, and well-designed policy interventions are key to overcoming vaccine hesitancy. Additionally, we consider the role of media and media approval in influencing public perceptions. Incorporating the COVID-19 experience from regions in India, this editorial provides actionable insights for healthcare professionals and policymakers.

## Introduction

Vaccine hesitancy, the reluctance or refusal to vaccinate despite vaccine availability, is influenced by misinformation, distrust in medical institutions, cultural beliefs, and personal risk assessments. The COVID-19 pandemic magnified these challenges, demonstrating the urgent need for targeted interventions. According to the World Health Organization (WHO), vaccine hesitancy was identified as one of the top 10 global health threats even before the pandemic^[2]^. During COVID-19, it became a leading barrier to achieving herd immunity in several regions. WHO data revealed significant discrepancies in COVID-19 vaccine uptake, with some low- and middle-income countries (LMICs) reporting coverage rates as low as 30%–40% in 2021 despite vaccine availability^[3]^. In India, for instance, misinformation on social media fueled vaccine resistance, contributing to avoidable morbidity and mortality. Moreover, vaccine hesitancy in some contexts was driven by legitimate concerns over the safety and efficacy of certain vaccines, particularly those that had received emergency use authorizations without long-term trial data^[4]^.

**Figure 1.**
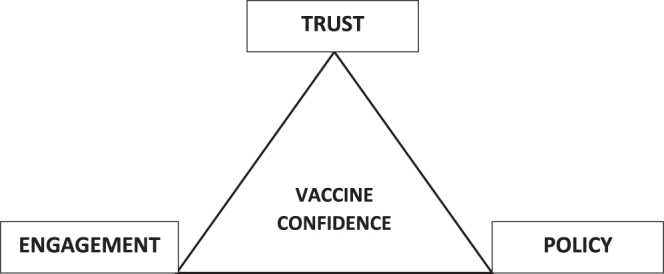
The TEP triad: A dynamic model for cultivating confidence during pandemics.

While public health agencies have responded with various strategies, there remains a need to understand what interventions are most effective in diverse socio-cultural contexts. Hence, this article examines key drivers of vaccine hesitancy and offers a framework for addressing vaccine hesitancy. We draw upon the Trust-Engagement-Policy (TEP) model to guide this editorial. The TEP model, comprising Trust, Engagement, and Policy, offers a dynamic, ethics-grounded approach to public health response during pandemics. Unlike traditional linear models that emphasize top-down information dissemination or behaviorist compliance (such as the Health Belief Model or Risk Communication frameworks), TEP highlights public trust’s reciprocal and iterative nature. Trust is not merely an outcome but a condition that shapes and is shaped by public engagement and policy decisions. Engagement in this triad involves participatory processes that enable communities to question, co-create, and adapt health strategies. Policy, in turn, must remain reflexive, responsive to the evolving trust landscape and the lived experiences of diverse populations. This framework emphasizes mutual reinforcement: trust enables deeper engagement, which informs better policy, which can consolidate or erode trust depending on how it is experienced. The TEP triad, as shown in Figure 1, is therefore not a static model but a dynamic feedback loop for sustaining confidence in public health systems during crises.

## Drivers of vaccine hesitancy: vaccine hesitancy

### Misinformation, social media impact, and vaccine approval concerns

As a primary challenge to building trust, misinformation, particularly on social media, erodes confidence in vaccination by distorting facts and amplifying fears. Studies indicate that delays in transparent communication contribute to the spread of misinformation, which fosters fear and vaccine refusal^[5]^. During the COVID-19 pandemic, rumors and conspiracy theories spread rapidly through social media, leading to public backlash against healthcare workers (HCWs) in various regions, including India. Additionally, Hesitancy in specific communities was exacerbated by concerns over the rapid development and approval of certain vaccines, which were perceived as insufficiently tested.

Additionally, public concern about the speed of vaccine development and the use of emergency use authorizations (EUAs) was not unfounded, particularly given the absence of long-term safety data during the early rollout phase. Comparative studies show that countries using vaccines with full regulatory approval (e.g. Pfizer-BioNTech in the U.S. and EU) reported higher initial public trust levels than those relying primarily on EUA vaccines with limited published data (for instance, Covaxin in India or Sputnik V in Russia during early phases)^[6]^. In India, survey data from early 2021 indicated that only about 40% of the population strongly believed in government-approved vaccines (locally made). This rate gradually improved as more data were released and endorsement by health professionals increased. This highlights the essential role of transparent, staged communication, not just about efficacy but also the scientific rigor and ethical review behind approval processes. Without such clarity, rapid approvals risk reinforcing public skepticism and undermining confidence even in highly effective vaccines.

### Community mistrust and alternative remedies

Effective engagement is critical in addressing long-standing community mistrust, especially in populations where traditional beliefs or historical injustices shape health behaviors. Recent data highlight the scope of public mistrust during pandemics. For instance, a WHO global survey in 2021 reported that nearly 38% of respondents expressed hesitancy toward COVID-19 vaccines, citing misinformation, lack of trust in government, and fear of side effects as major concerns^[7]^. Similarly, a CDC-led study found that in the United States, public trust in official pandemic messaging declined by 17% between early 2020 and late 2021, particularly among marginalized communities^[8]^. In many parts of India and other LMICs, especially during the early waves of COVID-19, communities turned to traditional herbal mixtures as perceived immune boosters^[9]^ in response to skepticism toward allopathic interventions. For example, substances such as Giloy (Tinospora cordifolia), Ashwagandha, and concoctions involving turmeric, ginger, and neem were widely promoted and consumed. While some herbs may have immunomodulatory effects, their unsupervised or excessive use poses health risks. A review by Subramani and Sathiyarajeswaran^[10]^ found that several commonly used herbal remedies had anticoagulant properties that, when consumed alongside or in place of prescribed medications, contributed to complications such as prolonged bleeding. Similarly, in North India, adverse events were noted where unregulated herbal supplement intake caused interactions with prescribed anticoagulants, resulting in extended clotting times and hemorrhagic episodes. The absence of formal guidance, poor regulation, and widespread distrust of mainstream medical messaging exacerbated these outcomes. Such cases underline the need for culturally sensitive engagement strategies and clear, respectful communication from health authorities to counter misinformation without alienating traditional belief systems.

### HCW distrust

Both trust and engagement are essential when addressing vaccine hesitancy among HCWs, who must feel respected, informed, and supported to serve as advocates. A significant challenge during the COVID-19 pandemic was the perception among HCWs that they were being used as “test subjects” for new vaccines. Studies highlight that HCWs in India reported feeling pressured to vaccinate without adequate information, leading to hesitancy. Furthermore, differences in vaccine brands available to HCWs, some perceived as less effective or lacking comprehensive safety data, contributed to their hesitancy. Addressing these concerns through structured training programs and transparent policymaking can boost confidence among frontline workers^[6]^.

### The role of policy interventions

Robust and transparent policy structures are necessary to institutionalize trust and engagement strategies, ensuring sustained vaccine confidence at the population level. Policy interventions play a crucial role in mitigating vaccine hesitancy. Evidence from past vaccination campaigns suggests that strategies such as establishing conveniently located vaccination clinics, offering financial incentives, and enforcing workplace vaccination mandates have increased vaccine uptake. In India, targeted campaigns in rural areas, backed by mobile vaccination units, significantly improved coverage during COVID-19^[5]^. Additionally, ensuring that vaccine approval processes are rigorous and well-communicated to the public can enhance trust in immunization efforts.

Furthermore, the underlying causes contributing to vaccine hesitancy and reduced vaccination rates include healthcare provider knowledge gaps, inadequate communication skills, systemic barriers, and misinformation influenced by cultural beliefs. Healthcare providers may lack up-to-date knowledge on immunization schedules, vaccine indications, and contraindications, limiting their ability to educate patients^[11]^. Even when knowledgeable, some healthcare providers struggle with communication skills, making it challenging to convey the importance of vaccines persuasively. Additionally, systemic barriers such as time constraints during patient visits, limited access to immunization records, and the absence of reminder systems hinder effective vaccine delivery. Moreover, widespread misinformation and deeply ingrained cultural or religious beliefs shape provider and patient perceptions, exacerbating vaccine hesitancy.

Research has consistently shown that exposure to even brief amounts of vaccine misinformation, sometimes as little as 5–10 minutes, can significantly reduce an individual’s willingness to vaccinate^[6,7,12]^. Broader knowledge gaps among patients and healthcare providers alike compound this impact. For instance, in one national study, 73% of physicians cited their patients’ limited understanding of disease prevention as a key barrier to vaccine acceptance^[12]^. These studies emphasize the importance of effective communication in countering misinformation and addressing baseline knowledge deficits. Without clear and accessible information, patients may fall back on cultural beliefs or online narratives that undermine trust in vaccination. Therefore, equipping healthcare providers with up-to-date knowledge and communication skills becomes vital in translating scientific guidance into public confidence.

**Figure 2.**
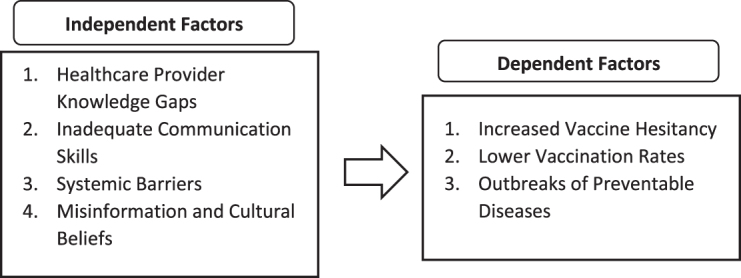
Contributing causes and outcomes of vaccine hesitancy on public health.

Additionally, the consequences of low vaccination coverage are evident in real-world cases, such as the 2019 measles outbreak in Samoa, which resulted in at least 83 deaths, primarily among young children, after vaccine coverage dropped from 84% to 31% due to misinformation and a temporary suspension of vaccination programs^[13]^. These statistics highlight the urgent need for comprehensive healthcare provider training, effective communication strategies, and systemic interventions to enhance vaccine uptake and prevent disease outbreaks in future pandemics. Furthermore, the consequences of inadequate training and information regarding vaccination include increased vaccine hesitancy, lower vaccination rates, and outbreaks of preventable diseases. A lack of proper training among healthcare providers can lead to misinformation or insufficient information being conveyed to patients, fostering vaccine hesitancy. Additionally, inadequate knowledge and training may cause providers to miss opportunities to recommend or administer vaccines, ultimately decreasing vaccination coverage. As a result, reduced vaccination rates can lead to the resurgence of preventable diseases, posing significant public health risks (Fig. 2).

### Regional insights from India and others

India’s experience during the COVID-19 pandemic offers powerful case studies that reveal the multifaceted nature of vaccine hesitancy and the effectiveness of context-sensitive interventions. In rural regions, misinformation, often spread via social media, intensifies resistance to vaccination and, at times, incites hostility toward HCWs. Traditional beliefs and widespread reliance on herbal remedies further complicated the situation, occasionally resulting in adverse health outcomes when such practices conflicted with medical advice^[10]^. Among healthcare professionals, mistrust was fueled, particularly in cases where vaccine brands varied in perceived efficacy and lacked transparent safety data^[6]^. At the policy level, India implemented localized awareness campaigns and deployed mobile vaccination units, which improved vaccine uptake in underserved areas^[5]^. These region-specific experiences showcase the importance of aligning communication, engagement, and policy strategies with local socio-cultural dynamics, an approach central to the TEP model.

One particularly impactful example was India’s CoWIN-enabled rural vaccination drive in mid-2021, which integrated mobile units, Accredited Social Health Activist (ASHA)-led outreach, and real-time data tracking to enhance coverage. In Maharashtra’s tribal Nandurbar district, where initial vaccine acceptance was below 30%, a community engagement initiative involving village panchayats and culturally adapted messaging increased vaccine coverage to over 95%^[4]^. Similarly, in Uttar Pradesh, ASHA workers conducted door-to-door counselling and flexible policy measures, such as pop-up vaccination sites at religious gatherings, further boosting uptake among hesitant groups. These outcomes demonstrate the tangible benefits of TEP-aligned strategies: community-based trust-building, locally relevant engagement, and adaptive policy implementation can significantly improve vaccine acceptance, particularly in communities facing systemic barriers and health inequities.

While the Indian context offers vital lessons, parallel strategies from other regions reinforce the universality of the TEP triad. In Sierra Leone during the Ebola crisis, local religious leaders and traditional healers were actively enlisted to convey accurate health information, contributing to increased public compliance and vaccine acceptance. In New Zealand, the government’s pandemic response emphasized transparency and empathetic communication, with the Prime Minister holding regular briefings framed in accessible language, resulting in some of the highest levels of public trust recorded globally during COVID-19. Meanwhile, early community mobilization efforts in Vietnam, such as neighborhood health checkpoints and participatory risk mapping, allowed for rapid containment with minimal top-down enforcement^[14]^. These cases affirm that pandemic responses rooted in trust-building, inclusive engagement, and adaptive policymaking can yield practical and ethical outcomes across varying governance styles and resource settings.

### TEP model

To systematically address vaccine hesitancy, we propose the TEP Model, which consists of three key components: Trust, built through transparent, science-backed communication; Engagement, which leverages community partnerships to strengthen local vaccine advocacy; and Policy, which ensures equitable vaccine access while addressing concerns about vaccine quality and approval processes. By integrating these elements into public health initiatives, the TEP Model can help shift vaccine attitudes from skepticism to confidence, ultimately improving vaccination uptake and public trust. Furthermore, to deepen the conceptual foundation of the TEP model, it is essential to examine how each component functions individually and interacts systemically.

Trust is the cornerstone of vaccine acceptance and is fostered through consistent, transparent, and empathetic communication from health authorities. This trust is in scientific facts, institutional integrity, and intentions. On the other hand, engagement builds on this trust by creating participatory spaces where communities, especially marginalized or skeptical groups, can voice concerns and receive context-specific responses. Engagement mechanisms may include dialogues with religious or traditional leaders, local language campaigns, and feedback loops that allow public input to shape messaging and strategy. Policy, as the structural component, reinforces trust and engagement by institutionalizing inclusive and evidence-based practices. Policies that ensure equitable vaccine distribution, protect against coercion, and enhance the visibility of safety oversight bodies can legitimize public health efforts. The interplay between these elements is dynamic: trust enables meaningful engagement, which informs responsive policy, while good policy sustains trust and engagement over time. The TEP model provides a circular and adaptive framework for transforming pandemic skepticism into durable public confidence.

**Figure 3.**
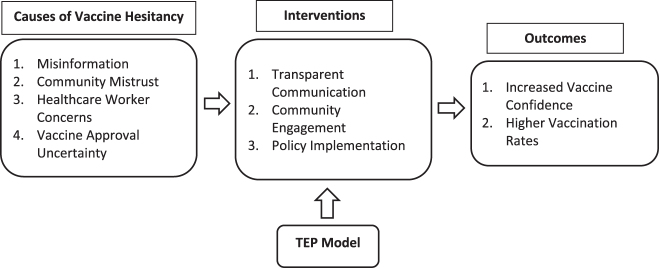
Trust-engagement-policy model.

Moreover, the TEP model (Fig. 3) builds upon existing conceptual frameworks, including the widely recognized World Health Organization (WHO, 2016) “3Cs” model, which attributes vaccine hesitancy to three core factors: Confidence (in vaccine safety and health authorities), Complacency (low perceived disease risk), and Convenience (barriers to access). While the 3Cs model effectively identifies the root causes of hesitancy, the TEP model advances this understanding by operationalizing solutions. For example, “confidence” in the 3Cs is addressed through transparent communication under the Trust pillar in TEP; “complacency” is mitigated through engagement that contextualizes disease risk within community values and experiences; and “convenience” aligns with Policy mechanisms that facilitate equitable access and logistical support. By reframing the problem from descriptive diagnosis (3Cs) to prescriptive strategy (TEP), the model presented here is uniquely suited to guide response efforts during fast-moving public health crises like pandemics, where actionable, scalable solutions are urgently needed.

Figure 3 shows a graphical representation illustrating the relationship between misinformation, community mistrust, HCW perceptions, vaccine approval concerns, and policy interventions. Additionally, to support the practical application of the TEP model in clinical settings, we propose a brief checklist that healthcare providers can use to identify and address vaccine hesitancy during routine patient encounters. This tool integrates the pillars of Trust, Engagement, and Policy into a simple framework, promoting evidence-based, empathetic, and actionable communication (Table 1).

**Table 1:** Checklist for healthcare providers: addressing vaccine hesitancy using the TEP model

TEP pillar	Actionable steps	Prompt questions/actions
Trust	Share transparent, evidence-based information about vaccine safety, efficacy, and side effects.	Would you like me to explain how this vaccine was tested and approved?
Engagement	Acknowledge cultural beliefs, ask about prior experiences, and validate concerns respectfully.	What have you heard about the vaccine in your community or from friends/family?
Policy awareness	Inform patients about eligibility, access points, and rights (e.g. free vaccines, post-vaccination support).	Did you know you can get the vaccine at the local clinic this weekend at no cost?

This checklist for healthcare providers addresses vaccine hesitancy across diverse settings. This tool will be used flexibly during individual patient consultations, group education sessions, or broader community outreach initiatives. By providing a structured approach, the checklist helps providers systematically build rapport with hesitant individuals, identify the root causes of their concerns, and channel communication strategies accordingly. It encourages empathetic dialogue, culturally sensitive engagement, and transparent information sharing to foster trust. Additionally, the checklist guides informed decision-making, enabling providers to clarify vaccine benefits, address misconceptions, and connect individuals with accessible vaccination services. This tool can support consistent, patient-centered discussions that align with public health goals while respecting individual autonomy in resource-limited settings or time-constrained encounters.

### Operationalizing the TEP checklist: guidance and barriers

The authors propose strategies sensitive to local contexts, infrastructure limitations, and governance capacities to operationalize the TEP checklist effectively. One important step is the establishment of Public Health Listening Units (PHLUs), ideally embedded within district health offices and staffed by multilingual personnel who can monitor public sentiment across digital and offline platforms, such as WhatsApp groups, community radio, and panchayat meetings. These units should routinely synthesize findings into actionable insights for state-level policy reviews. Another foundational practice is conducting trust audits prior to policy implementation. These can be spearheaded through collaborations between local NGOs and academic institutions using tools such as mobile polling apps and focus group discussions to map out which communities trust, and why.

Utilizing existing community health networks also plays a crucial role. In the Indian context, for example, ASHAs, Anganwadi workers, and faith-based volunteers already hold community credibility. They can act as two-way communicators between public health authorities and the public, provided they receive additional training in pandemic literacy, rumor mitigation, and digital consent. In parallel, all emergency communications and digital interventions should undergo ethics review to identify potential harms, such as exclusion of those without digital access or the perception of coercion. These ethics committees should be diverse, incorporating professionals, laypersons, and communication experts to ensure cultural and contextual appropriateness. Finally, the development of feedback-responsive policy templates is vital. Such templates should be designed for iteration, incorporating real-time trust metrics and public concerns, for example, allowing vaccine deployment plans to shift in response to fluctuating uptake rates or the emergence of misinformation hotspots. Together, these measures anchor the TEP checklist not as a static tool but as a flexible, community-responsive framework for building confidence in public health systems.

### Some perceived potential barriers and mitigation strategies

The TEP checklist’s practical implementation also demands a clear understanding of potential barriers and corresponding mitigation strategies. One common challenge is the presence of infrastructure gaps, particularly in low-resource settings where digital connectivity or healthcare infrastructure is weak^[15]^. These limitations can obstruct the rollout of PHLUs or the execution of trust audits. However, partnering with civil society organizations and using low-tech solutions, such as printed surveys, street theatre, or local storytelling forums, can bridge these divides and ensure community input remains central. Another critical barrier is political interference, which can erode public trust when health messaging becomes politicized or inconsistent. To counter this, establishing independent oversight mechanisms and transparent communication protocols can help insulate public health messaging from political agendas.

A further concern is burnout among frontline health workers, who are often tasked with additional engagement responsibilities during health crises. Without formal recognition, compensation, or support, this can lead to demotivation and attrition. Integrating engagement into their official role descriptions, backed by regular stipends or incentives, is essential to sustaining their involvement. Finally, privacy risks must be addressed proactively, especially when collecting trust-related or sentiment data. To avoid surveillance concerns or misuse, all data should be anonymized and gathered through consent-driven processes, ideally reviewed by ethics committees. By anticipating these challenges and embedding adaptive, community-centered safeguards, the TEP checklist becomes a theoretical model and a practical and responsive tool for building public confidence in health systems.

## Conclusion

Overcoming vaccine hesitancy requires a multifaceted and multi-sectoral approach integrating clear communication, community engagement, and policy-driven strategies. This article highlights the importance of region-specific interventions supported by empirical evidence. Moving forward, governments and healthcare institutions must prioritize transparent dialogue, rigorous vaccine approval standards, and evidence-based policymaking to foster vaccine confidence and ensure robust immunization programs. Future research should empirically test and refine the TEP model across diverse socio-cultural and political contexts. Mixed-methods studies could examine how trust, engagement, and policy interact in real-time during vaccine rollouts, identifying which elements are most influential in different populations. Longitudinal studies may also be valuable in assessing how interventions aligned with the TEP model affect vaccine confidence over time.

Additionally, implementation research can explore the feasibility and scalability of community-led engagement strategies and policy frameworks rooted in transparency. Comparative analyses between regions that adopted TEP-aligned approaches and those that did not could further validate the model’s utility. Hence, integrating the TEP model into future public health planning may strengthen pandemic preparedness and improve routine immunization programs globally.

## Data Availability

Information provided is publicly available.
